# A European survey on awareness of post-surgical adhesions among gynaecological surgeons

**DOI:** 10.1007/s10397-013-0824-2

**Published:** 2013-11-27

**Authors:** Markus Wallwiener, Philippe Robert Koninckx, Andreas Hackethal, Hans Brölmann, Per Lundorff, Michal Mara, Arnaud Wattiez, Rudy Leon De Wilde, for The Anti-Adhesions in Gynecology Expert Panel (ANGEL)

**Affiliations:** 1 Department of Obstetrics and Gynaecology, University of Heidelberg, Heidelberg, Germany; 2 University Hospital Gasthuisberg, Katholieke Universiteit, Leuven, Belgium; 3 Queensland Centre for Gynaecological Cancer, Queensland Brisbane, Australia; 4 VU University, Amsterdam, The Netherlands; 5 Gynecologic Clinic, Private Hospital Molholm, Vejle, Denmark; 6 Charles University, Prague, Czech Republic; 7 Hôpital de Hautepierre, Strasbourg, France; 8 Klinik für Frauenheilkunde, Geburtshilfe und Gynäkologische Onkologie, Universitätsklinik für Gynäkologie, Pius-Hospital, University Oldenburg, Oldenburg, Germany

**Keywords:** Post-surgical adhesions, Gynaecological surgery, Awareness, Prevention

## Abstract

The present survey was conducted among gynaecological surgeons from several European countries to assess the actual knowledge and practice related to post-surgical adhesions and measures for reduction. From September 1, 2012 to February 6, 2013, gynaecological surgeons were invited to answer an 18-item online questionnaire accessible through the ESGE website. This questionnaire contained eight questions on care settings and surgical practice and ten questions on adhesion formation and adhesion reduction. Four hundred fourteen surgeons participated; 70.8 % agreed that adhesions are a source of major morbidity. About half of them declared that adhesions represented an important part of their daily medical and surgical work. About two thirds informed their patients about the risk of adhesion. Most cited causes of adhesions were abdominal infections and extensive tissue trauma, and endometriosis and myomectomy surgery. Fewer surgeons expected adhesion formation after laparoscopy (18.9 %) than after laparotomy (40.8 %); 60 % knew the surgical techniques recommended to reduce adhesions; only 44.3 % used adhesion-reduction agents on a regular basis. This survey gives a broad picture of adhesion awareness amongst European gynaecological surgeons, mainly from Germany and the UK. The participants had a good knowledge of factors causing adhesions. Knowledge of surgical techniques recommended and use of anti-adhesion agents developed to reduce adhesions need to be improved.

## Background

Post-surgical adhesions—abnormal fibrous connections developing between the peritoneum and organs as a sequel to surgical trauma—are the most frequent complication of abdominal surgery and may represent one of the greatest unmet medical needs of the moment [1].

Yet, many surgeons are still not aware of the extent of the problem and its serious consequences, such as chronic pelvic pain and small bowel obstruction. In addition, post-surgical adhesions are a frequent cause of dyspareunia and secondary infertility.

In a previous survey conducted among gynaecological surgeons in German hospitals, adhesions were believed to develop in 15 % of cases after laparoscopy and 40 % after laparoscopy [2].

In symptomatic patients, removal of post-surgical adhesions requires a new surgical intervention (adhesiolysis). However, adhesiolysis is often followed by adhesion reformation. In this situation, earlier precautions aiming to prevent post-surgical adhesions are of paramount importance.

Developments in adhesion-reduction strategies and new agents now offer a realistic possibility of reducing the risk of adhesions forming and, thus, may improve the outcomes for patients and the associated onward burden.

Based on the fact that for an adhesion to form, there must be a prolonged contact between two areas of injury, two measures are currently recommended to minimise post-surgical adhesions: good surgical practice with minimal tissue trauma, and in addition, anti-adhesion agents used intra-operatively to minimise contact between injured parts of the peritoneum and an adjacent organ [3]. Both measures aim to reduce the abnormal healing process that results in the formation of adhesions.

Epidemiological data have demonstrated that despite these advances in prevention, the burden of adhesion-related complications has not changed [4–8].

In this context, the actual knowledge and practice of gynaecological surgeons with regard to this complication of their interventions was assessed in several European countries. A survey was conducted in order to document the awareness of the risk of post-surgical adhesions amongst gynaecological surgeons, the knowledge of measures to be taken to minimise this complication of surgery, the surgical procedures likely to cause extensive adhesions, the information given to the patients about the risk of post-surgical adhesions during the consenting process, and subsequently the actual practice regarding the prevention of adhesions.

## Methods

Gynaecological surgeons were recruited through the micro-website dedicated to post-surgical adhesions developed by the European Society for Gynaecological Endoscopy (ESGE) (http://www.esge.org/index.php?option=com_surveyforce&view=survey&Itemid=101). Both members and non-members of the ESGE could participate.

Website visitors were invited to fill in an 18-item online questionnaire (Appendix). On top of the questionnaire, the micro-website featured a printable information leaflet for patients about the risk of adhesions and a pictorial version of the ESGE expert consensus position on the prevention of post-surgical adhesions [9].

No financial incentives were proposed to the survey participants.

Due to the nature of the survey, the statistics were purely descriptive and expressed in percentages. Means and standard deviations, medians, minimum, and maximum were calculated where applicable. These calculations were not corrected for missing data.

## Results

Between September 1, 2012 and February 6, 2013, 233 gynaecological surgeons completed the whole questionnaire; another 181 participated in the survey but left at least one question unanswered.

Out of the 414 participants, 356 (86 %) downloaded the ESGE expert consensus position paper on adhesions.

### Care settings and levels of activity

**Fig. 1:**
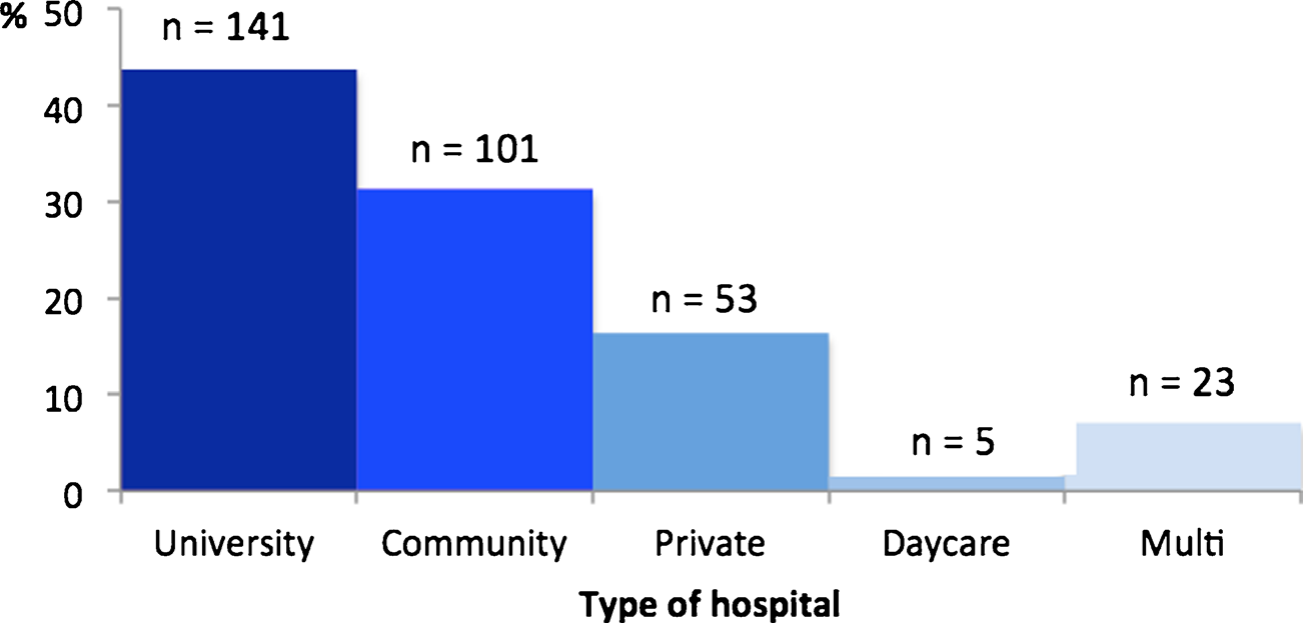
Distribution of survey respondents per type of hospitals

Although the survey participants worked in a variety of care settings, a majority (75 %) worked exclusively or partially in a university or a community hospital (Fig. 1). The two main countries represented were the UK (20.6 % of participants) and Germany (20.0 %), followed by Italy (16.2 %) and the Netherlands (7.5 %).

Owing to the 265 participants who answered this question, the mean number of laparotomic, laparoscopic, and vaginal interventions performed per gynaecology department in 2010 was 1,213, 606, and 389, respectively. However, the actual numbers reported for each department varied widely (Table 1).

**Table 1:** Mean and median numbers of interventions performed in 2010 in the gynaecology departments of the survey respondents (all participating countries)

Intervention type	Mean ± SD	Median
Laparotomic	1,213 ± 1,719	700
Laparoscopic	606 ± 710	380
Vaginal	389 ± 1,033	200

**Fig. 2:**
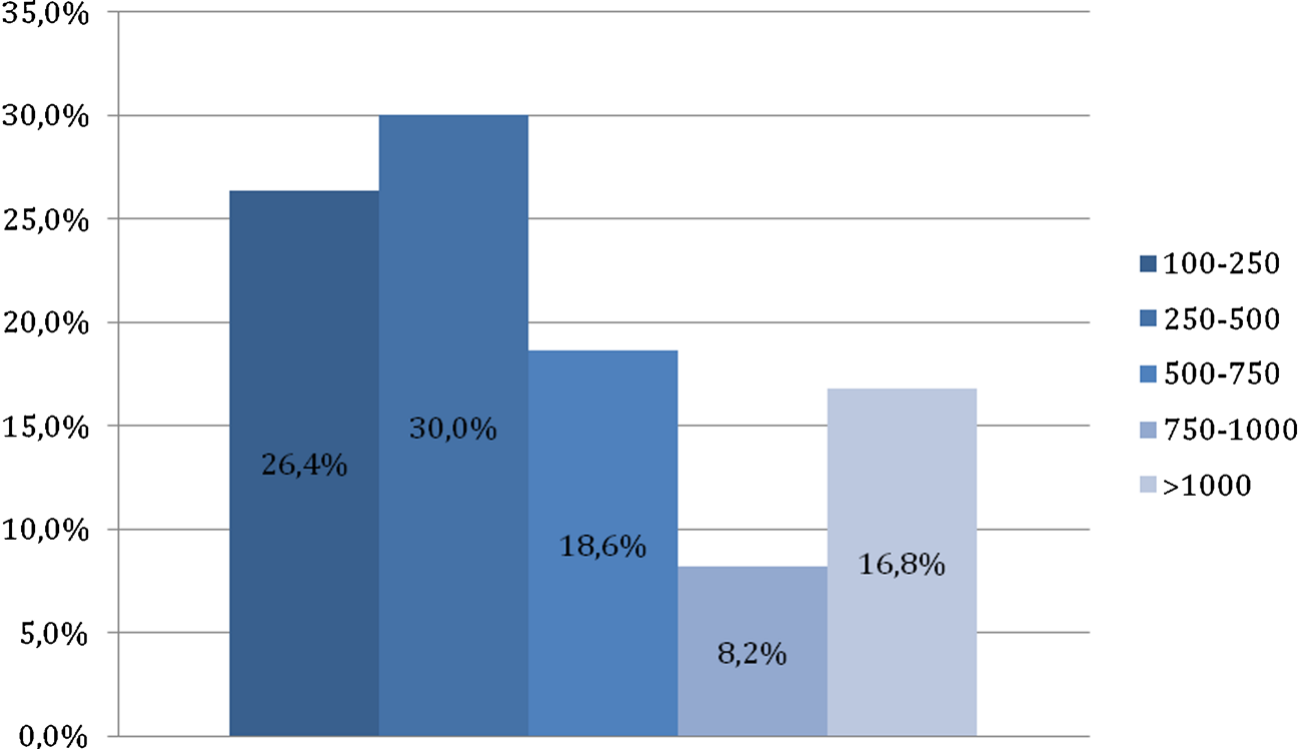
Number of laparoscopic interventions performed by each gynaecological surgeon

The number of laparoscopic interventions performed by each gynaecological surgeon during the previous 5 years was also variable (Fig. 2).

Table 2 summarizes the number of surgical interventions performed in 2010, per hospital type, in the two main participating countries (UK and Germany).

**Table 2:** Summary of the number of surgical interventions performed in 2010 per hospital type: Germany and UK data

Type of hospital	Country	Percentage of participants providing data on number of interventions % (*n*/*N*)	Laparotomies mean number ± SD	Laparoscopies mean number ± SD	Vaginal route mean number ± SD
University hospital	Germany	66.6 (24/36)	1,236.7 ± 1,344.8	1,624.7 ± 1,730.0	998.7 ± 1,390.2
	UK	87.5 (42/48)	1,649.4 ± 1,086.6	827.6 ± 512.9	437.4 ± 333.2
Community hospital	Germany	83.3 (25/30)	409.5 ± 302.2	750.0 ± 589.7	298.0 ±264.9
	UK	78.9 (15/19)	1,518.2 ± 1,647.2	622.7 ± 562.7	265.9 ± 208.6
Private hospital	Germany	78.5 (11/14)	155.9 ±132.5	780.3 ± 819.8	383.1 ± 796.4
	UK	87.5 (7/8)	1,155.8 ± 1,214.1	1,192.7 ± 1,425.4	748.3 ± 1,163.7
Daycare hospital	Germany	100 (2/2)	0.0 ±0.0	1,650.0 ±1,202.	1,200.0 ± 1,131.4
	UK	0	–	–	–

**Fig. 3:**
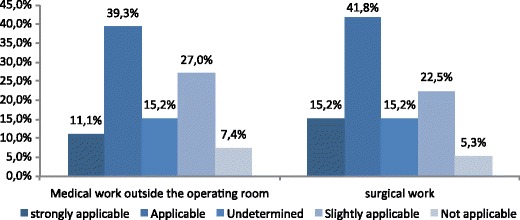
Importance of patients with post-surgical adhesions in a gynaecologist’s daily work

Among 253 responders, 70.8 % agreed that post-surgical adhesions are a source of major morbidity. They were 50.4 and 57.0 %, respectively, to declare that patients with adhesions represented an important to very important part of their daily medical work outside of the operating room and of their daily surgical work (Fig. 3).

### Patient consenting

Out of 244 responders to the inquiry regarding the daily practice of consenting their patients about adhesions, 64.3 % declared they provide information about the risk of adhesion formation. Further, 65.6 % declared to provide information regarding possible complications of adhesions and 52.5 % declared to provide information regarding treatment options for adhesions (Table 3).

**Table 3:** Selected results of the present survey, presented after Hackethal et al [2]

Entry	% of participants
Adhesions considered as a major source of morbidity	70.8
Adhesions considered as an important part of daily medical work	50.4
Adhesions considered as an important part of daily surgical work	50.7
Estimated incidence of adhesions post-laparotomy	40.8
Estimated incidence of adhesions post-laparoscopy	18.9
Patients informed of risk of adhesions during consenting	64.3
Regular use of anti-adhesion agents	44.3
Anti-adhesion agents considered as cost-effective	77.5
Anti-adhesion agents considered as too expensive	71.6
Anti-adhesion agents considered as insufficiently refunded	75.8
Consider themselves as well informed about adhesions	60.0
Source of adhesion knowledge	
Scientific publications	85.6
Personal experience	82.6
Discussions with colleagues	75.8
Continuous medical education	84.7
Consensus paper	66.5
ESGE conferences	61.5

### Surgical procedures leading to intra-abdominal adhesion formation

For 40.8 ± 22.1 % of the survey participants, laparotomic interventions were associated with a risk of post-surgical adhesions; they were fewer to associate this risk with laparoscopic interventions (18.9 ± 16.3 %), vaginal surgery (22.1 ± 17.1 %) or natural orifice transluminal endoscopic surgery (NOTES) (17.6 ± 16.9 %). The difference between laparotomy and laparoscopy was independent from the type of surgical intervention considered (Table 4).

**Table 4:** The type of surgery in benign conditions leading to intra-abdominal adhesions with the estimated likelihood on a scale from 0 (unlikely) to 4 (highly likely)

Type of surgery	Median score ± SD of 5-point Likert rating scale	
	Laparotomy	Laparoscopy
Endometriosis surgery	3.6 ± 0.6	2.8 ± 0.8
Myomectomy	3.4 ± 0.7	2.6 ± 0.9
Adhesiolysis	3.3 ± 0.7	2.5 ± 0.9
Adnexal surgery	2.9 ± 0.8	2.6 ± 0.8
Hysterectomy	3.1 ± 0.6	2.0 ± 0.7
Ectopic pregnancy	2.2 ± 0.8	
Caesarean section	2.5 ± 0.8	

Among the different gynaecological operations, endometriosis surgery and myomectomy were thought to be the most likely to be associated with adhesions (Table 4). The risk was considered low with caesarean section and only occasionally associated with ectopic pregnancy, single port, and NOTES.

### Considerations regarding surgical adhesion induction

Table 5 indicates the characteristics thought to have a high impact on the formation of adhesions. Intra-abdominal infections and extensive tissue trauma were quoted as having the highest impact.

**Table 5:** Parameters influencing adhesion formation and the estimated likelihood on a scale from 0 (unlikely) to 4 (highly likely)

Characteristic	Median score ± SD of five-point Likert rating scale
Infections within abdomen	3.7 ± 0.7
Extensive tissue trauma	3.7 ± 0.6
Postoperative infections	3.6 ± 0.8
Previous surgeries	3.6 ± 0.6
Foreign body incompatibility	3.2 ± 1.0
Quantity of sutures/staples/meshes	3.2 ± 0.9
Blood in abdomen	3.2 ± 0.9
Extensive coagulation	3.2 ± 0.9
Chronic inflammatory bowel diseases	3.1 ± 1.0
Affinity to reduce wound healing	2.8 ± 0.9

Virtually all the gynaecological surgeons (94.8 % of 238 responders) considered that good surgical practice was important to prevent post-surgical adhesions. They were 60.5 and 55.3 %, respectively, to consider antiadhesive barriers and peritoneal conditioning as important.

**Fig. 4:**
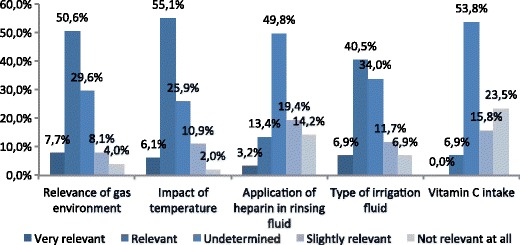
Significance of some aspects of the peritoneal conditioning in the adhesions prevention (*N* = 247)

The relevant elements of peritoneal conditioning identified by 247 respondents were temperature, gas environment, and the type of irrigation fluid (Fig. 4). Additional preparation of the rinsing fluid had an undetermined effect for heparin and for vitamin C (Fig. 4).

### Indications for surgical adhesiolysis

The main reasons for adhesioysis were symptoms (95.0 % of the responders), infertility (93.7 %), young age (73.5 %), and previous surgery (68.9 %); 53.4 % of the responders declared that adhesiolysis was performed in all patients.

### Awareness of anti-adhesion agents

The survey participants were asked whether they knew and utilized the currently available anti-adhesion agents. Although the formation of adhesions was a topic of major interest for 90.3 % of 236 responders, no single agent was known by more than 60 % of them; Ringer lactate was the anti-adhesive barrier most frequently used and additionally considered as most important anti-adhesive barrier (Table 6).

**Table 6:** Summary of different adhesion prophylaxis products, knowledge of their existence, use, and importance rated on a scale from 0 (do not know this agent) to 2 (used it within the last 6 months)

Adhesion prophylaxis products	Known (% of participants)	Used (% of participants)	Importance
Ringer lactate	53.8	38.2	1.3 ± 0.6
Adept/Icodextrin 4 %	55.5	26.5	1.1 ± 0.7
Interceed®	56.3	23.9	1.0 ± 0.7
Hyalobarrier Gel®	56.3	19.3	0.9 ± 0.7
Humidified/warm CO_2_	55.5	18.1	0.9 ± 0.7
Intercoat®	48.3	9.7	0.7 ± 0.6
SprayShield®	56.3	9.7	0.8 ± 0.6
Seprafilm®	63.8	4.6	0.6 ± 0.6

**Fig. 5:**
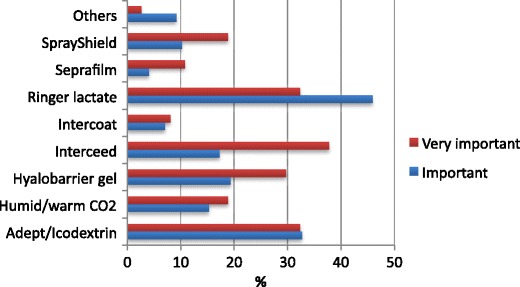
Use of adhesion-reduction agents within the six previous months, as a function of importance given to adhesions in daily surgical work

Anti-adhesion agents were used on a regular basis (at least twice in the previous month) by 44.3 % of 253 responders (Table 6). Figure 5 suggest that except for Ringer lactate, use of antiadhesive barriers was positively influenced by the importance given to adhesions in daily medical and surgical work.

For 77.5 % of 236 responders, adhesion prevention was deemed cost-effective because it eliminates further adhesion-related interventions. However, a majority declared that antiadhesive barriers are too expensive and insufficiently refunded by health insurance systems (71.6 and 75.8 %, respectively).

More than 60 % of the survey participants estimated they were adequately informed about the pathogenesis of adhesions and the techniques recommended and agents proposed to prevent adhesions.

Table 3 indicates the relative importance of sources of this knowledge.

### Intraoperative adhesion assessment

The criteria useful for a classification of the risk of adhesions in routine practice were the area coverage for 95.3 % of the 236 responders, the location for 93.2 %, the macroscopic evaluation for 92.4 %, the organs involved for 91.5 %, and the lysis characteristics for 79.7 %.

## Discussion

This survey reflects a strong interest of participating European gynaecological surgeons in post-surgical adhesions and their prevention measures. More than 90 % of participants declared their awareness on adhesions and over 95 % agreed that good surgical practice may reduce the formation of adhesions. In line with conventional knowledge, they were a majority to consider that laparoscopic interventions are associated with a much lower incidence of adhesions than laparatomic interventions, although strong evidence supporting this assertion is lacking.

The survey participants had a good knowledge on factors associated with a high risk of post-surgical adhesion formation, similar to those quoted in the literature [10]. Surgery for endometriosis was thought to be majorly associated with the formation of adhesions, followed by myomectomy, adhesiolysis, and adnexial surgery. These results were independent of the type of surgical approach, laparotomy or laparoscopy. However, the assumption that laparoscopic adnexal surgery was associated only occasionally with a limited risk of adhesion formation, would need to be confirmed by a wider scale study.

Most of our data are in agreement with those of a previous survey performed in 2010 among heads of gynaecological departments in Germany (Table 3) [2]. In particular, the estimated risks of post-surgical adhesions are similar in both surveys and confirm that laparoscopic procedures are commonly believed to be less adhesiogenic and cause fewer de novo adhesions compared to open surgery [11]. However, for complex laparoscopic procedures, the comparative risk of adhesion-related complications following open and laparoscopic gynaecological surgery is similar [5, 10].

The rate of information about post-surgical adhesions given to the patients (Table 3) was markedly lower in our survey than in the Hackethal survey [2]. Conversely, we report here a more frequent use of anti-adhesion agents (44.3 vs 22.0 %). Elucidating whether these differences are linked to the mode of recruitment of the two surveys (open to all gynaecological surgeons visiting the ESGE website or through a direct contact with the heads of gynaecological departments in Germany) is beyond the scope of the present work.

The data presented here suggest that efforts should be made to increase awareness of the risk of post-surgical adhesions and knowledge of the preventive measures. About one third of surgeons considered themselves as not adequately informed about the pathogenesis of adhesions and the preventive measures. Consistent with this finding, about 40 % ignored the existence of one or more of the antiadhesive barriers currently marketed and utilization of these agents was clearly sub-optimal.

Furthermore, we noted a distinct discrepancy between the knowledge of the existence of adhesion prophylaxis products of nearly more than half of the respondents (ranging from 48.3 to 63.8 % )compared to low percentage of participants routinely using barriers (ranging from 4.6 to 38.2 % regarding the usage in the last 6 months). Some products such as Seprafilm ® had an inverse ratio with the highest awareness (63.8 %) compared to low routine usage (only 4.6 %). In addition, barriers such as Icodextrin were rated as important by a large number of participants, despite the scientific evidence.

This could be explained by contortioned perception due to lack of awareness of scientific sources such as the ESGE consensus paper [9].

The fact that lactated Ringer’s solution was considered as the most frequently used prevention method and ranked as most important could be explained by cost-driven considerations due to a lack of reimbursement as well clearly shows the need for evidence based education.

There is also a need for improvement of patient information and consenting about the risk of post-surgical adhesions. It has been shown in a population of patients from Germany and the UK that less than 50 % were aware of adhesions and even fewer were informed about the possible complications of adhesions; 46 % of patients cited the surgeon lack of knowledge as the reason for not informing them [12]. Comparatively, the higher rate of patient information reported by our survey participants seems encouraging—but might be due to a selection bias: the majority of surgeons that volunteered to answer our questionnaire had probably a strong interest in adhesion-related issues.

Cost considerations may contribute to the limited regular use of antiadhesive barriers. These survey participants considered such barriers as too expensive and insufficiently refunded by health systems. These opinions were already expressed by the German survey participants [2]. Thus, regarding the economical impact of intraoperative utilization of antiadhesive barriers, there seems to be a gap between the opinion of gynaecological surgeons and that of decision-makers who shape national public health policies. While the former are sensitive to the potential long-term benefits of antiadhesive barriers, the latter are most probably motivated by immediate reduction of healthcare-related expenses. Furthermore, the evidence for the use of antiadhesion barriers is limited. Though, in experimental and clinical studies, adhesion reduction works in reducing adhesions, there is a lack of efficacy in terms of clinical benefits (i.e. reduction of pain and improved fertility).

Limitations of this survey should be taken into account when interpreting its results.

As all surveys, this one was based on self-reporting of information by the participants and the data were not censored. Many participants did not answer all questions and no methodology was planned to recover the missing data. Also, whether our survey describes accurately the opinions of the whole community of European gynaecological surgeons is questionable. However, the wide variation in the number of interventions performed would suggest that the participants were at least representative of the different levels of experience of European gynaecologists in current practice.

In summary, the present survey gives a broad picture of awareness of post-surgical adhesions and their reduction among European gynaecological surgeons. Results are generally encouraging but emphasize the necessity to continue educational activities in order to optimize the rate of practitioners applying the measures recommended to reduce this common complication of gynaecological surgery. In particular, a widespread dissemination of the field guidelines on the prevention of adhesions in gynaecological surgery published in 2012 [13] is warranted. An information leaflet has also been published to help surgeons inform their patients about the risk of adhesions, their potential complications, and their reduction measures [14].

Reducing the personal and economical burden of post-surgical adhesions should become a common goal for all gynaecological surgeons. The present survey shows that further efforts should be made to ensure that all women—in particular those wishing to conceive—can benefit from the solutions designed to reduce post-surgical adhesions and their complications.
